# Significant improvements in self-reported gastrointestinal tolerability, quality of life, patient satisfaction, and adherence with lopinavir/ritonavir tablet formulation compared with soft gel capsules

**DOI:** 10.1186/1742-6405-5-21

**Published:** 2008-09-17

**Authors:** Shannon Schrader, Susan K Chuck, Laurie W Rahn, Paras Parekh, Katherine G Emrich

**Affiliations:** 1 The Schrader Clinic, Houston, USA; 2 Abbott Virology, Abbott Laboratories, Abbott Park, USA

## Abstract

**Background:**

The tablet formulation of ritonavir-boosted lopinavir (LPV/r; Kaletra^®^) has many advantages over the soft gel capsule (SGC) formulation, including lower pill count, no refrigeration requirement, and no dietary restrictions. These advantages may help improve patient compliance and therefore increase adherence to treatment. However, there are limited data regarding patient preferences and only recently was the comparative efficacy and tolerability data of LPV/r SGC versus tablet formulation presented at an international conference. To address this deficit, we conducted a market research survey to assess potential tolerability benefits, patient satisfaction, changes in adherence, and formulation preference in patients switching from SGCs to the tablet formulation. Data from 332 patients who switched from LPV/r SGCs twice-daily (BID) to tablets BID and 41 patients who switched from LPV/r SGCs BID or once daily (QD) to tablets QD were analyzed.

**Results:**

Switching from SGCs to a tablet formulation of LPV/r was associated with increased patient satisfaction, tolerability and self-reported adherence to treatment; gastrointestinal side effects were reduced. In addition, respondents indicated that they preferred the tablet formulation to the SGC.

**Conclusion:**

The LPV/r tablet formulation provides HIV-infected patients with multiple benefits over the SGC in terms of tolerability and convenience. Additional assessments to further define the tolerability profile of the LPV/r tablet, including studies using once-daily dosing, are warranted.

## Introduction

Lopinavir/ritonavir (LPV/r, Kaletra^®^), a co-formulation of lopinavir (LPV) and ritonavir (RTV), is a protease inhibitor (PI) used in the treatment of HIV infection. The low dose of RTV in the LPV/r formulation increases LPV exposure by decreasing metabolism of LPV via inhibition of cytochrome P450 3A. RTV concentrations achieved are below therapeutic concentrations. LPV/r, dosed at 400/100 mg in the form of three soft gel capsules (SGCs) BID, has been shown to provide sustained virological response (over seven years) as well as excellent immunological responses in 61% of patients, with an on-treatment response rate of 95% (HIV RNA < 50 copies/mL) [1]. In addition, no primary PI resistance mutations or thymidine analog mutations were observed during the seven years of this study.

The original formulation of LPV/r was SGCs because the active ingredients are poorly soluble and have poor bioavailability when administered as an unformulated solid. While SGCs have been widely used in the US and elsewhere, there are some significant limitations:

• Pill count is high, requiring six capsules for the standard daily dose (LPV/r 800/200 mg).

• Similar to RTV capsules, LPV/r SGCs exposed to high temperatures are susceptible to softening and clumping and may become impossible to separate without breaking, which may result in loss of drug and subsequently inadequate drug exposure [2]. LPV/r SGCs must be refrigerated before dispensing to the patient and stored at room temperature by the patient.

• The SGCs must be taken with food, creating the potential for inter- and intra-patient variability.

• The SGCs are associated with short to medium-term gastrointestinal (GI) side effects, mostly nausea, vomiting, and diarrhea.

Several of these limitations are overcome with the recent reformulation using melt extrusion technology. The tablet formulation of LPV/r is bioequivalent to the SGC formulation at a dose of 800/200 mg with reduced pharmacokinetic variability [3]. The LPV/r tablet formulation is associated with a significantly reduced food effect, in that increasing meal calories and fat content does not significantly affect C_max _and AUC. The tablet formulation is also significantly more bioavailable than the SGC formulation when taken in a fasted state, which allows the flexibility to dose LPV/r tablets with or without food. Furthermore, the bioavailability of LPV is unaffected by acid-reducing agents irrespective of whether the SGC or tablet formulation is taken [4].

LPV/r was the first co-formulated antiretroviral to be formulated as a tablet by melt extrusion. This technology produces a solid dispersion (or solid suspension) whereby the LPV/r molecules are uniformly distributed throughout a hydrophilic polymeric matrix. This ensures that the drug is released at a consistent rate in the GI tract and allows for adequate bioavailability (previously unachievable with traditional tablet formulations). Advantages of the tablet formulation include a lower pill count (four tablets *versus *six SGCs per day), no refrigeration requirement, and no dietary restrictions.

The effectiveness of antiretroviral therapy (ART) depends on treatment efficacy as well as patient adherence. Barriers to adherence include adverse drug effects, pill count, dosing frequency, dietary/fluid requirements and the need for refrigerated storage. The LPV/r tablet formulation addresses some of these barriers and may improve treatment adherence.

There are limited data regarding patient preferences and only recently at the Conference on Retroviruses and Opportunistic Infections 2008 was the comparative efficacy and tolerability of LPV/r SGC vs. tablet formulation presented [5]. In this study, we report the results of a market research survey, which was designed to assess the differences in tolerability benefits, patient satisfaction, changes in adherence, and formulation preference in patients switching from LPV/r SGCs to tablets.

## Methods

The survey was a self-administered written questionnaire completed by patients at baseline and after switching from LPV/r SGCs to tablets. Patients were required to have a minimum of four weeks' experience taking each formulation prior to filling out each survey. Surveys were distributed with the help of 52 participating physicians across 20 states plus D.C. and completed by patients in their doctors' offices/clinics while waiting for their regularly scheduled appointments. Staff at the physicians' offices were instructed to maintain patient privacy by having respondents insert completed surveys into envelopes before returning them; the staff then mailed them to the research organization managing the project. Physicians received an honorarium for each completed survey that was received by the research organization. Packets of pre- and post-switch questionnaires (both English and Spanish versions), with instructions for their distribution, were sent to physicians in early October 2005. The deadline for returning baseline surveys was May 2006.

### Questionnaire design

Surveys were designed by Abbott Marketing Research. Additional input from advisors was used to attain an appropriate language and reading level (grade 5) for patients. The majority of questions were identical in the baseline and follow-up surveys for a longitudinal pre-*versus *post-switch analysis. Five comparative questions were added to the follow-up questionnaire so that respondents could make a direct assessment of SGCs *versus *tablets.

### Statistical analysis

The Student's t-test was used for the analysis of continuous data; nominal data were analyzed using Chi-squared and Fisher's exact tests. While no power calculation was completed, the sample size is large, including over 300 respondents.

## Results

### Patient demographics

The demographics of survey respondents are shown in Table 1. There was a predominance of males and the majority of respondents were over 35 years of age. More than 80% of the respondents had received ART for more than one year, received LPV/r SGCs for more than one year, and received tablets for less than three months. All respondents in the analysis had received SGC and tablet formulations for at least four weeks each.

**Table 1:** Respondent demographics

%	**Patients switching from SGCs BID to Tablets BID (%)**	**Patients switching from SGCs BID or QD to Tablets QD (%)**
**Male/Female**	85/15	76/24

**Age:**		
**> 45 years**	46	34
**35–44 years**	41	39
**< 35 years**	13	27

**Duration of antiviral therapy**		
**≥ 5 years**	59	41
**1–5 years**	31	49
**< 1 year**	10	10

**Duration of LPV/r therapy**		
**SGC > 1 year**	82	80
**Tablet < 3 months**	89	81

### Improved tolerability and fewer side effects

#### Patients switched from SGCs BID to tablets BID

**Figure 1:**
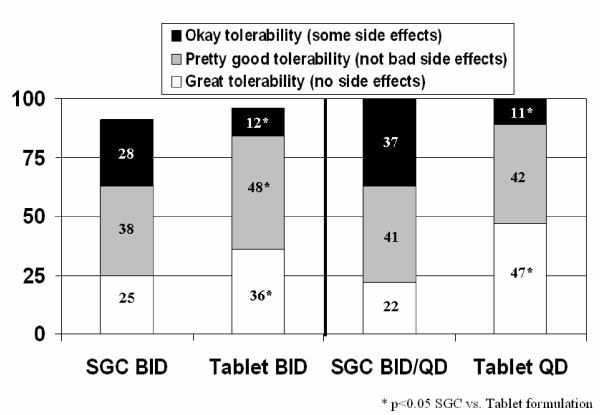
Tolerability reported by respondents switched to tablets BID or QD.

Three hundred and thirty-two patients who completed linked questionnaires (pre- and post-switch) had switched from SGCs BID to tablets BID. Before switching to the tablet formulation, 60% of respondents reported that they were "very satisfied" or "extremely satisfied" with their treatment. This proportion increased to 80% (*p *< 0.05) after these respondents switched to tablets BID. There was also a significant increase in the proportion of respondents describing the tolerability of their treatment, with respect to side effects, as "great" or "pretty good" when they switched from SGCs BID to tablets BID (63% vs. 84%; *p *< 0.05) [Figure 1].

Respondents reported a significant improvement in diarrhea after switching from SGCs to tablets; 82% of respondents reported either no diarrhea or an improvement in diarrhea after this switch. This included a 21% increase in the proportion of respondents who indicated no or rare diarrhea (*p *< 0.05). Only 3% of respondents reported "severe" diarrhea while receiving the tablet formulation compared with 12% receiving SGCs (*p *< 0.05). Antidiarrheal use (3+ times per week) was reduced by half after respondents switched to the tablet formulation (*p *< 0.05).

Significantly fewer respondents reported bloating, pain, or gas in the stomach when they were switched to the tablet formulation, and those who did reported diminished frequency: an additional 12% of respondents indicated no occurrence or rare occurrence (*p *< 0.05). Only 5% reported "severe" episodes of bloating, pain, or gas in the stomach after the switch to the tablet formulation, compared with 8% when receiving the SGC formulation (*p *< 0.10).

#### Patients switched from SGCs BID or QD to tablets QD

Forty-one patients who completed pre- and post-switch questionnaires had switched from SGCs BID (n = 20) or QD (n = 21) to tablets QD. Before switching, 73% of respondents were "very satisfied" or "extremely satisfied" with their treatment; 64% rated tolerability, with respect to side effects, as "great" or "pretty good". After switching to tablets QD, 90% of respondents were "very satisfied" or "extremely satisfied" with their treatment and 89% rated tolerability of treatment as "great" or "pretty good" [Figure 1]. This represented an increase of 17% in patient satisfaction (*p *< 0.05) and an increase of 26% for tolerability (*p *< 0.05).

In the 20 respondents who switched from SGCs BID to tablets QD, there was a 33% absolute increase in the number who were "extremely satisfied" with their treatment (from 30% pre-switch to 63% post-switch; *p *< 0.05). For those switching from SGCs QD to tablets QD, there was a 43% absolute increase in the proportion of respondents who were "extremely satisfied" (from 14% pre-switch to 57% post-switch; *p *< 0.05). There was also a significant increase in the proportion of respondents who had "pretty good" or "great" tolerability when switched to tablets QD: a 24% absolute increase for those switching from SGCs BID to tablets QD (from 70% pre-switch to 94% post-switch; *p *< 0.05) and a 29% absolute increase for those switching from SGCs QD to tablets QD (from 56% pre-switch to 85% post switch; *p *< 0.05).

Overall, GI side effects improved after respondents were switched to the LPV/r tablet formulation. After switching from SGCs BID or QD to tablets QD, 78% of respondents reported no diarrhea or had improvements in diarrhea; more respondents who had switched from SGCs QD (81%) than from SGCs BID (74%) reported improvements. Reports of bloating, pain, or gas in the stomach also decreased when respondents were switched from SGCs to tablets, and the prevalence of nausea decreased from 27% to 5% (*p *< 0.05). There were no "severe" episodes of diarrhea, nausea, and bloating/gas with the tablet formulation compared with 0%, 8% and 6%, respectively, with the SGC formulation. The need for antidiarrheal medications was decreased; 80% of respondents reported rare or no use with tablets compared to 56% with SGCs (*p *< 0.05).

### Greater self-reported adherence

#### Patients switched from SGCs BID to tablets BID

After patients switched from SGCs to tablets, the mean number of self-reported missed doses per week decreased from 1.25 to 0.71 (*p *< 0.05), equivalent to an improvement in adherence from 91% to 95%. In addition, more respondents indicated "not missing doses in the last week" after switching to the tablet formulation. Taking fewer pills per dose than prescribed was reported for 5% of SGC doses and only 1% of tablet doses (*p *< 0.05), with no differences between ethnic groups.

**Figure 2:**
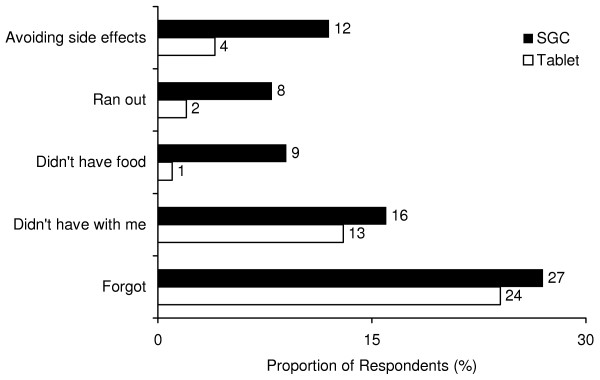
Reasons for non-adherence in patients switched from SGCs BID to tablets BID.

Significantly fewer respondents cited "avoiding side effects", "ran out", and "didn't have food" as reasons for non-adherence (*p *< 0.05) [Figure 2].

#### Patients switched from SGCs BID or QD to tablets QD

The mean number of self-reported missed doses per week decreased from 0.49 to 0.24 after patients were switched from SGCs BID or QD to tablets QD, equivalent to an improvement in adherence from 93% to 97%. Taking fewer pills per dose than prescribed was reported for 2.1% of SGC doses and 0.4% of tablet doses, with no differences between ethnic groups. A greater proportion of respondents indicated "not missing doses in the last week" after switching to tablets (90% on tablets vs. 75% on SGCs). "Avoiding side effects" was cited by 5% of respondents receiving SGCs as the reason for non-adherence; when these patients switched to tablets, this reason was no longer cited.

### Patients prefer the tablet to the SGC

#### Patients switched from SGCs BID to tablets BID

Eighty-eight percent of respondents who switched from SGCs BID to tablets BID preferred the tablet to the SGC formulation, 9% had no preference, and the remaining 3% preferred the SGC.

Benefits of the tablet formulation over the SGC that the respondents "liked" were: "don't need to refrigerate" (67%), "fewer pills" (61%) and "don't have to take with food" (41%).

Respondents were asked to rate their quality of life over the last four weeks as "very bad, could hardly be worse", "pretty bad", "good and bad parts about equal", "pretty good" or "very well, could hardly be better". A large proportion (73%) of respondents reported better quality of life when taking the tablet; only 2% of respondents reported a worse quality of life. There was also a significant shift in respondents reporting quality of life that had "good and bad parts about equal" to "pretty good" after switching to the tablet formulation (21% and 48% before switching, to 14% and 58% after switching, respectively; *p *< 0.05).

#### Patients switched from SGCs BID or QD to tablets QD

Ninety-three percent of respondents who switched from SGCs BID or QD to tablets QD preferred the tablet to the SGC; 5% had no preference between the two formulations and 2.5% preferred the SGC. Benefits of the tablet that were "liked" by the respondents included: "once daily dosing" (73%), "fewer pills" (73%), "don't have to take with food" (68%) and "don't need to refrigerate" (65%).

Quality of life over the last four weeks (rated by respondents as "very bad, could hardly be worse", "pretty bad", "good and bad parts about equal", "pretty good" or "very well, could hardly be better") was improved when respondents switched to the tablet formulation: 73% of respondents reported an improvement and 2% reported worsening with tablets dosed QD. An additional 12% of respondents reported that they were "very well, could hardly be better"; these respondents had previously reported "good and bad parts about equal" and "pretty good".

### Ethnic differences

Data from the 332 patients who switched from SGCs BID to tablets BID were analyzed according to ethnicity. Twenty percent of respondents were Hispanic, 37% were Black, 41% were Caucasian, and 2% were other ethnicities.

The proportions of respondents indicating better side effects with the tablet formulation were similar: 74% of Caucasian, 85% of Black, and 76% of Hispanic respondents. Differences among ethnic groups in reductions of the frequency and severity of diarrhea are shown in Table 2; there was significant improvement in all groups. There was also a trend for a decrease in antidiarrheal use; however, this only reached statistical significance in the Caucasian group (RR 0.72, SGC vs. tablet; *p *< 0.05).

**Table 2:** Ethnic differences in diarrheal side effects in respondents switching from BID SGCs to BID tablets

	BID Respondents (n = 332)	White (n = 136)	Black (n = 123)	Hispanic (n = 67)
Relative Risk (Tablets vs. SGCs)				

Antidiarrheal use (more than "rarely use")	0.73*	0.72*	0.95	0.65
Diarrhea frequency 3+ per week	0.60*	0.68*	0.52*	0.39*
Respondent self-defined "severe" diarrhea	0.28*	0.47*	0.13*	0.17*

Proportion of respondents (Tablets vs. SGCs)				

Respondent self-defined "severe" diarrhea	3.3% vs. 11.7%*	7% vs. 15%*	1% vs. 8%*	2% vs. 12%*

* *p *< 0.05

A similar proportion of each ethnic group preferred the tablet over the SGC (86%–89%), experienced "great" to "pretty good" tolerability (82%–87%), and felt "very satisfied" or "extremely satisfied" (79%–85%) with their treatment. In addition, the rank order of cited tablet benefits was the same across ethnic groups: "don't need to refrigerate" > "fewer pills" > "don't have to take with food". Significantly more SGC doses were taken without food by Black respondents (21%) than Caucasians (12%), *p *< 0.05.

The Caucasian group showed a high adherence rate on SGCs (95%), leaving little room for improvement when switched to tablets. Hence, the 96% adherence rate after switching was not statistically significant. However, mean weekly adherence rates did significantly improve in Hispanic (from 89% on SGCs to 96% on tablets; *p *< 0.05) and Black respondents (from 88% on SGCs to 93% on tablets; *p *< 0.05), who had lower mean adherence rates on SGCs compared with Caucasians. A significant improvement in complete adherence (defined as "not missing doses in the last week") was observed in Hispanic respondents (from 55% on SGCs to 72% on tablets; *p *< 0.05). On SGCs, significantly more Black respondents (18%) than Caucasians (5%) indicated missing doses in an attempt to avoid adverse effects. A comparison of the ethnic groups using the tablet formulation did not show any difference in missing doses in an attempt to avoid adverse effects.

The number of doses missed in an attempt to avoid adverse events was significantly reduced with switching from SGC to tablets in Black and Hispanic respondents (*p *< 0.05), with a non-significant decrease also noted for Caucasians.

## Discussion

In this study, pre- and post-switch patient surveys revealed that the new tablet formulation of LPV/r is preferred over SGC formulation, is better tolerated, has fewer side effects, and is associated with greater adherence than the original SGC formulation. These results support the data from three studies that examined switching patients from LPV/r SGC to tablet formulation. The first study involved indigent AIDS patients and demonstrated that four weeks following the formulation switch there were greater patient preference and satisfaction with LPV/r tablet, and significant improvements in bowel habits that were maintained through Week 12. There was a positive impact on subjects' overall well-being, as measured by Global Condition Improvement Questionnaire; however, the positive changes in other quality of life measures (MOS-HIV PHS, MOS-HIV MHS, ASDM, and CES-D) were not statistically significant [6]. The second study was a sub-study of the IMANI-2 LPV/r single agent trial. The key findings were a reduction or elimination of diarrheal adverse events, improved general health and ability to function in role at work or in the home, improved self-reported ease of taking medication, and high satisfaction and preference for LPV/r tablet formulation [7]. The third study evaluated improvements in quality of life associated with switching from LPV/r SGC to tablet in an African American cohort. The study showed 96% of patients preferred the LPV/r tablet formulation, and modest improvements in general health, mental health and role function as measured by MOS-HIV. The majority of patients indicated their quality of life was improved because of a lack of need to refrigerate tablets (80%), fewer pills (76%), and the removal of food restrictions (60%) [8].

The majority of survey respondents (89%) in the current analysis had been switched from LPV/r 400/100 mg SGC BID to tablets BID. There were significant increases in patient satisfaction and tolerability after the switch to the tablet formulation. There were also improvements in the proportion of respondents reporting diarrhea. Fewer respondents reported severe diarrhea with a significant reduction in the frequency of antidiarrheal use and side effects of bloating, pain, or gas in the stomach.

A smaller proportion of survey respondents (11%) had switched to a QD tablet regimen, either from LPV/r 800/200 mg SGC QD or 400/100 mg SGC BID. In 2005, the FDA approved QD dosing of LPV/r 800/200 mg in treatment-naïve adults. The approval was based on an analysis of two randomized, controlled clinical trials in which the safety and efficacy of LPV/r QD (800/400 mg) versus twice-daily (400/100 mg) doses were compared in treatment-naïve patients [9-11]. Abbott Study 418 evaluated LPV/r SGC QD versus BID dosing and showed an increase in the incidence or severity of GI side effects for QD compared with the BID regimen [11]. This is in contrast to the findings of the largest study of LPV/r tablet formulation, Abbott Study 730, where LPV/r SGC QD and BID dosing were compared to LPV/r tablet QD and BID dosing in 664 subjects [5]. Abbott Study 730 concluded that QD and BID dosing have similar overall safety and tolerability. In our study, respondents that were switched from LPV/r SGC to QD LPV/r tablet reported increased satisfaction and tolerability after switching to the QD regimen regardless of whether the pre-switch SGC regimen was dosed QD and BID. There were greater improvements reported by survey respondents switching from the QD SGC regimen to the QD tablet regimen [5].

Lower mean LPV trough concentrations were reported in Abbott Study 056 and Study 418 with LPV/r 800/200 mg QD dosing (3.62 mcg/mL and 4.37 mcg/mL, respectively) compared to LPV/r 400/100 mg BID dosing [10,11]. However, antiretroviral activity was not affected. A recent analysis of 5 clinical trials of LPV/r dosed QD and BID concluded that there is no significant association between mean LPV trough concentration and virologic response in naïve patients [12]. Additionally, trough LPV concentrations < 1 mcg/mL were not associated with virologic failure. Currently, QD dosing of LPV/r is not recommended for treatment-experienced patients due to a lack of clinical safety and efficacy data. However, the efficacy and safety of LPV/r QD versus LPV/r BID in treatment-experienced patients is currently being investigated in Abbott Study 802, a large, randomized 48-week clinical trial [13].

Previous studies of antiretroviral choice have shown that patients find lack of side effects more important than convenience (e.g. pill count, dosing frequency, etc.) and this may impact adherence [14,15]. In this study, reduction in side effects impacted patient-reported adherence. When patients switched from SGCs to tablets, adherence (as assessed by number of self-reported missed doses per week and the number of respondents who indicated "not missing doses in the last week") improved and significantly fewer respondents cited "avoiding side effects" as a reason for non-adherence. An analysis of adherence rates by ethnic groups with LPV/r tablet has not been reported previously. Importantly, Hispanic and Black respondents reported significantly improved adherence. Racial differences in non-adherence in order to "avoid side effects" were no longer evident after the formulation switch.

In addition to reducing GI side effects, the tablet formulation provides greater convenience because it reduces the pill count (four tablets per day as opposed to six SGCs), can be tolerated as a QD regimen, and does not need to be refrigerated. Also, while the SGC has to be taken with food for adequate bioavailability, there is limited effect of food on LPV pharmacokinetics with the tablet formulation [3]. The vast majority of respondents (88% of those who switched to a BID regimen and 93% of those who switched to a QD regimen) preferred the tablet to the SGC because of these factors. Seventy-five percent of respondents who switched to the QD regimen indicated they "liked" taking LPV/r just once a day. Another reason for greater patient preference for the tablet formulation is improvement in quality of life, observed in 73% of respondents after switching from SGCs to tablets.

While our sample did not include adequate numbers of females for a meaningful analysis of gender differences, the effect of gender on the safety and efficacy of LPV/r was evaluated in Abbott Study 730. Similar rates of moderate or severe diarrhea, nausea, and vomiting were observed for males and females [16]. Abbott Study 730 also evaluated the safety of LPV/r tablets in whites *versus *non-whites and reported that moderate or severe diarrhea rates were similar for whites and the overall study population, while non-whites rates were lower [17]. Our study compared more ethnic groups (Caucasians, Blacks, and Hispanics) and also included a comparison of LPV/r SGC to tablet. Tolerability and satisfaction were found to significantly improve across all ethnic groups (Hispanic, Black, and Caucasian) with the switch to LPV/r tablets. The rates of "severe" diarrhea were reduced in all ethnic groups with Caucasians having the greatest reduction in antidiarrheal use.

We are aware that this study is based on market research and that the survey used is not a validated instrument or quality of life measure. However, it does provide information regarding patient perceptions and preferences of LPV/r therapy.

## Conclusion

It is clear from this study that patients prefer the new LPV/r tablet formulation due to improvements in side effects and greater convenience. Further studies are required to prospectively compare the LPV/r tablet formulation with other drug regimens. Until now, all clinical trial comparisons *versus *other PIs and EFV have been made with LPV/r SGC [18-23]. Emerging data from the TITAN and CASTLE studies include a mixture of LPV/r SGC and tablet due to the studies being started prior to LPV/r tablet receiving FDA approval. In the ARTEMIS study, only 2% of patients solely used LPV/r tablets, so further data to discern tolerability differences between other PIs, EFV, and LPV/r tablets are still needed [24-26].

## Abbreviations

ART: antiretroviral therapy; BID: twice-daily; GI: gastrointestinal; LPV: lopinavir; LPV/r: lopinavir/ritonavir; PI: protease inhibitor; QD: once-daily; RTV: ritonavir; SGC: soft gel capsule.

## Competing interests

Shannon Schrader has participated in the following companies' speaker bureaus: Abbott, GSK, BMS, Gilead, Roche, and Tibotec. Susan K. Chuck, Laurie W. Rahn, Paras Parekh, and Katherine G. Emrich are Abbott employees and own Abbott stock or options.

## Authors' contributions

SS participated in the analysis and interpretation of the data and the development of the manuscript. SKC participated in the conception and design of the study, analysis and interpretation of the data, and development of the manuscript. LWR participated in the conception and design of the study, and the analysis and interpretation of the data. PP participated in the conception and design of the study. KGE participated in the conception and design of the study, and the analysis and interpretation of the data. All authors read and approved the final manuscript.
